# Population Seroprevalence Study after a West Nile Virus Lineage 2 Epidemic, Greece, 2010

**DOI:** 10.1371/journal.pone.0080432

**Published:** 2013-11-18

**Authors:** Georgia A. F. Ladbury, Magda Gavana, Kostas Danis, Anna Papa, Dimitris Papamichail, Spiros Mourelatos, Sandra Gewehr, George Theocharopoulos, Stefanos Bonovas, Alexis Benos, Takis Panagiotopoulos

**Affiliations:** 1 European Programme for Intervention Epidemiology Training (EPIET), European Centre for Disease Prevention and Control (ECDC), Stockholm, Sweden; 2 Dutch National Institute for Public Health and the Environment (RIVM), Bilthoven, The Netherlands; 3 Aristotle University of Thessaloniki, Thessaloniki, Greece; 4 Hellenic Centre for Disease Control and Prevention (KEELPNO), Athens, Greece; 5 National School of Public Health, Athens, Greece; 6 Ecodevelopment S.A., Thessaloniki, Greece; Metabiota, United States of America

## Abstract

**Introduction:**

During summer 2010, 262 human cases including 35 deaths from West Nile virus (WNV) infection were reported from Central Macedonia, Greece. Evidence from mosquitoes, birds and blood donors demonstrated that the epidemic was caused by WNV lineage 2, which until recently was considered of low virulence. We conducted a household seroprevalence study to estimate the spread of infection in the population during the epidemic, ascertain the relationship of infection to clinical disease, and identify risk factors for infection.

**Methods:**

We used a two-stage cluster design to select a random sample of residents aged ≥18 years in the outbreak epicentre. We collected demographic, medical, and risk factor data using standard questionnaires and environmental checklists, and tested serum samples for presence of WNV IgG and IgM antibodies using ELISA.

**Results:**

Overall, 723 individuals participated in the study, and 644 blood samples were available. Weighted seropositivity for IgG antibodies was 5.8% (95% CI: 3.8–8.6; n=41). We estimated that about 1 in 130 (1:141 to 1:124) infected individuals developed WNV neuroinvasive disease, and approximately 18% had clinical manifestations attributable to their infection. Risk factors for infection reflected high exposure to mosquitoes; rural residents were particularly at risk (prevalence ratio: 8.2, 95% CI: 1.1–58.7).

**Discussion:**

This study adds to the evidence that WNV lineage 2 strains can cause significant illness, demonstrating ratios of infection to clinical disease similar to those found previously for WNV lineage 1.

## Introduction

West Nile virus (WNV) is a flavivirus primarily transmitted by *Culex* mosquitoes. Wild birds are the natural host, while horses and humans are dead-end hosts [1]. WNV strains form at least two main lineages: lineage 1 (L1), which has a wide geographic distribution ranging from Europe, Africa, the Middle East and America and was responsible for previous outbreaks in the Mediterranean and North America, and lineage 2 (L2), which had only been found in sub-Saharan Africa and Madagascar until 2004, when it was isolated in a Hungarian goshawk with neurological disease [2,3]. Most WNV L1 human infections are asymptomatic, but clinical infections occur and range from mild, self-limiting illness to severe neuroinvasive disease, with a 12–20% case fatality in patients manifesting encephalitis or meningoencephalitis [1]. In the past, it was hypothesized that WNV L2 caused only mild disease in humans, but recent evidence suggests that virulent and neuroinvasive L2 strains may be circulating [3-5]. 

**Figure 1:**
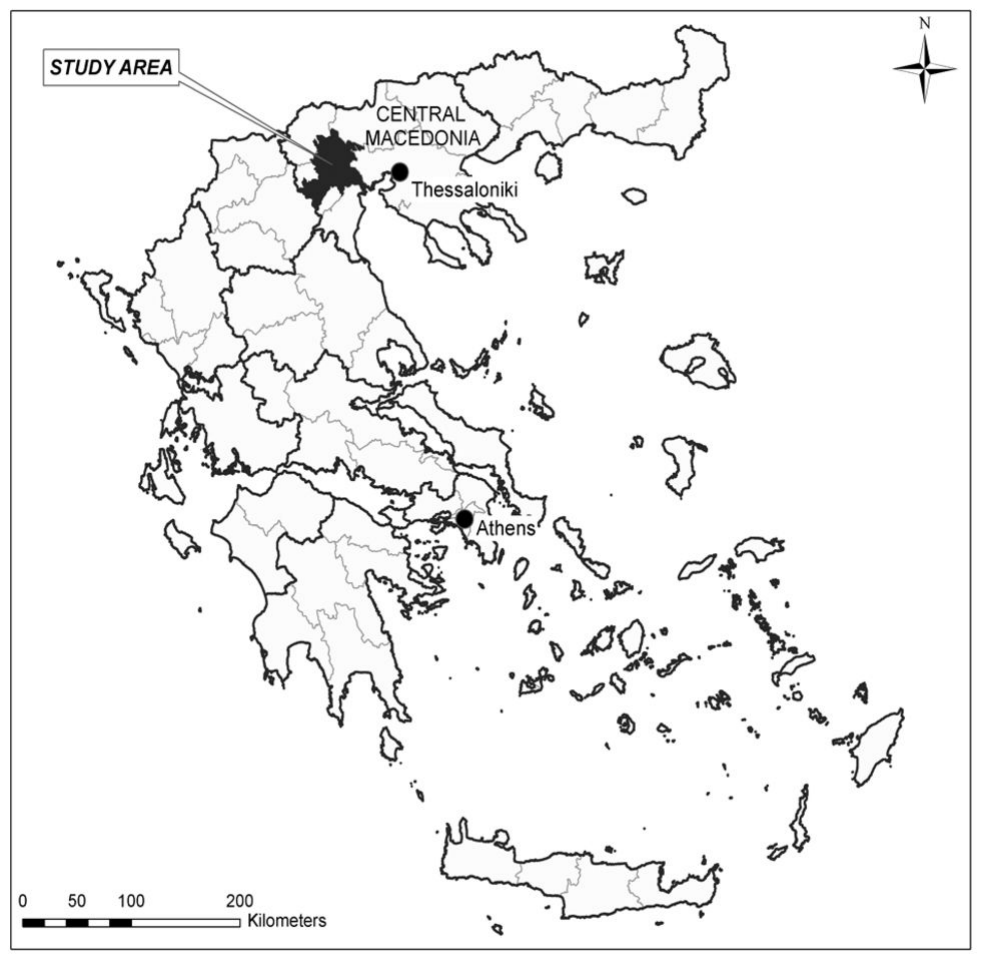
Map of Greece with study area. (See enclosed figure)

In summer 2010, an epidemic of WNV infection occurred in Central Macedonia in northern Greece, starting in a major wetland area and resting point for migratory birds (Figure 1). This was the first time that WNV clinical disease was reported in Greece, with 262 confirmed cases, 197 of which were cases of WNV neuroinvasive disease (WNND), and including 35 fatalities [6]. Since 2010, all WNV sequences obtained from affected areas in Greece were of lineage 2 [7-12]. In 2010, WNV L2 was isolated from a blood donor who presented with mild symptoms two days after donation, while similar sequences were obtained from three *Culex* mosquito pools and a wild bird [7-9]. In 2011–2012, WNV L2 was isolated from five *Culex* mosquito pools and three sentinel chickens [9-12]. Genetic characterisation of the Greek WNV L2 strain (Nea Santa-Greece-2010) has shown that the virus is closely related to the Hungarian goshawk isolate, and contains an amino-acid substitution (H_249_P) in non-structural protein 3 that has been previously associated with increased virulence in L1 strains [13,14]. In the past few years, outbreaks related to WNV L2 strains have also been reported from other countries within the European region [15,16].

Previous studies of WNV L1 outbreaks had indicated that 75–80% of infections are asymptomatic, 20–25% present as West Nile fever (WNF) [17,18], and <1% go on to develop WNND [17,19-22], with risk factors for infection including exposure to mosquitoes, birds, stagnant water, and outdoor activities [17,21-23]. Whether this knowledge could be extrapolated to L2 outbreaks was unclear. We therefore conducted a household-based seroprevalence study to estimate the spread of infection in the population during the epidemic, ascertain the relationship of infection to clinical disease, and identify risk factors for infection.

## Methods

### Study population and sampling design

The study population was defined as the adult population (≥ 18 years) resident in 84 townships (towns or villages) in the lowland areas of Imathia and Pella prefectures, Central Macedonia, where the highest incidence of WNND had occurred during the 2010 outbreak (27 and 28 cases per 100,000 population, respectively) [6]. We used two-stage cluster sampling to select a random sample of the study population. A cluster comprised six randomly distributed households in a township, selected using maps of residential blocks or houses. In the first stage, we randomly selected clusters from the study area using probability proportional to township population size (according to 2001 census data) [24,25]. In the second stage, all residents of selected households aged 18 or over were invited to participate. We calculated that 60 clusters would be required to obtain seroprevalence estimates with 2.5% precision and a 95% confidence level, assuming 2.4 adults per household (2001 census data) [24,25], response rate of 65–70%, design effect of 2, and seroprevalence of 5% (somewhat higher than had been found in some previous WNV seroprevalence studies to ensure a sufficient sample size) [17,21]. 

### Data collection

Selected households were recruited into the study by fieldwork coordinators. In case of non-inhabitation, non-answer, or refusal to participate, the clockwise neighbouring household was approached as a substitute. At least three visits at different times of the day were made to selected households prior to substitution. Fieldwork was undertaken by trained field researchers between November 25 and December 22, 2010, 14–18 weeks after the epidemic peak and 7–11 weeks after the last case was reported.

All participants were interviewed at their household using a standard questionnaire concerning demographic characteristics, medical history, and exposure to potential risk or protective factors. One participant per household answered a second questionnaire concerning household-level characteristics, such as presence of screens on windows and potential sources of standing water. We tested the questionnaires through a pilot study involving 20 purposively selected persons from the study area. For rural (population <2,000) and semi-urban (population 2,000–20,000) areas, we collected additional information regarding local environmental characteristics through structured observation using checklists. 

Participants were asked to provide a 3ml blood sample on the day of interview. Standard precautions were taken regarding phlebotomy, transport and cold storage of samples. Serum samples were tested for WNV-specific IgM and IgG using ELISA (WNV IgM μ-capture DxSelect and WNV IgG DxSelect, Focus Diagnostics, Cypress, CA) using cut-offs according to the manufacturer’s instructions. An avidity test using urea 6M was performed in the IgG-positive samples for probable differentiation between acute and non-acute infection; avidity index higher than 40% was suggestive of non-acute (>3 months) infection. Positive samples were also tested for antibodies to two other flaviviruses, tick-borne encephalitis virus (TBEV) (TBE/FSME IgM and TBE/FSME IgG; IBL International Gmbh, Hamburg, Germany) and dengue fever virus (DENV) (IgM Capture DxSelect and IgG DxSelect; Focus Diagnostics) to rule out these infections.

### Statistical analysis

We performed statistical analysis adjusting for cluster design and weighting by age and area of residence, as younger age-groups and people living in cities were under-represented in the sample compared to 2001 census data (table S1). We analyzed data in Stata (Stata Corporation, Texas, USA, version 11).

#### Seroprevalence and relationship of West Nile virus neuroinvasive disease to infection

We calculated seroprevalence estimates for both IgG and IgM. To determine the ratio of WNND to infection, we compared the number of cases reported from the study area during the epidemic to the estimated number of infections given the IgG seroprevalence we found and the population size in the study area according to 2001 census data [24,25]. To estimate the total number of infections that occurred nationally, we extrapolated from this ratio taking into account the total number of WNND cases reported in Greece during the 2010 epidemic (n=197) [6], assuming that persons <18 years of age had a similar risk of infection as the adult population. 

Sensitivity analysis: we repeated the calculation of IgG seroprevalence and WNND-to-infection ratio classifying as IgG-negative persons who were IgG-positive/IgM-negative and had low outlier values of IgG index (n=4, Figure S1).

#### Occurrence of West Nile fever

To investigate the relationship between seropositivity and WNF, we categorised participants according to whether or not they had experienced at least three of the following WNF indicator symptoms during the same episode (from June to September 2010): (1) fever, (2) chills, (3) headache, (4) myalgia or arthralgia, (5) skin rash, (6) generalised weakness, and (7) ocular pain [18]. The weighted proportion of symptomatic seronegative persons was subtracted from that of symptomatic IgG-positive persons in order to ascertain the risk of developing symptoms that was attributable to WNV infection. Confidence intervals of attributable risks were calculated using standard formulae [26], based on sampling errors of proportions derived from weighted analysis. We repeated this calculation for subjects with two or more indicator symptoms. We used the average of these two attributable risk results to estimate the proportion of persons manifesting symptomatic disease after WNV infection.

#### Factors associated with seropositivity

To explore associations between exposure variables and IgG seropositivity, we calculated prevalence ratios (PR) from the weighted proportions in the exposed and unexposed groups. We included exposure variables with a PR>2 or <0.5 or a p-value <0.05 in a logistic regression analysis. To simplify the models, variables were removed one at a time depending on significance testing (p<0.05) using the likelihood ratio test. We then used binomial regression to derive adjusted PRs using all the variables of the final logistic regression model. Potential interactions were also examined. We additionally performed a sub-analysis of the rural and semi-urban data to include the environmental variables from these areas. 

### Ethics

All participants provided written informed consent. Ethical approval was obtained from The Bioethics Committee of the National School of Public Health, Greece.

## Results

### Study participation

A total of 360 households participated in the study; 216 (60%) were those initially selected, and 144 (40%) were substitutions. Approximately two thirds of substitutions (69%) were due to non-inhabitation or non-answer of the original household, and one third (31%) due to refusal to participate. Of 850 eligible residents, 723 (85%) participated, and 644 (76%) provided a suitable blood sample.

Local environmental information was available for all 234 households in rural and semi-urban areas, corresponding to 586 eligible residents, and 491 (84%) study participants; blood results were available for 443 (76%) of these partcipants.

### Seroprevalence and relationship of West Nile virus neuroinvasive disease to infection

WNV IgG antibodies were detected in 41 out of 644 persons tested (weighted seroprevalence 5.8%; 95% CI: 3.8–8.6); in 15 of them WNV IgM antibodies were also detected (weighted seroprevalence 1.9%; 95% CI: 1.0–3.5). Seroprevalence was highest in residents of rural areas, and IgG-positive as well as IgM-positive participants tended to be older than those who were seronegative (table 1, table S2).

**Table 1:** West Nile virus IgG seroprevalence by gender, age-group, and area of residence.

		**WNV IgG-positive**			
		**n/N**	% (95% CI)*	Prevalence ratio (95% CI)*	**P-value**
**Gender**	Female	23/344	5.6 (3.5–8.7)	Reference	
	Male	18/300	6.0 (3.3–10.5)	1.07 (0.57–2.00)	0.835
**Age (years)**	18–39	4/161	2.7 (1.0–7.1)	Reference	
	40–59	14/238	5.7 (3.1–10.1)	2.10 (0.82–5.34)	0.120
	60–69	11/103	10.6 (6.1–17.9)	3.95 (1.33–11.69)	0.013
	70+	12/142	8.7 (4.8–15.3)	3.22 (1.14–9.09)	0.027
**Area of residence†**	Urban	3/201	1.4 (0.3–6.9)	Reference	
	Semi-urban	12/198	5.3 (2.8–9.8)	3.84 (0.68–21.68)	0.127
	Rural	26/245	10.0 (6.5–15.3)	7.28 (1.37–38.78)	0.020
**Total**		41/644	5.8 (3.8–8.6)		

WNND: West Nile neuroinvasive disease 95% CI: 95% confidence interval

* Proportions and confidence intervals are weighted by age and area of residence, and adjusted for cluster design.

† Area of residence: urban: >20,000 inhabitants; semi-urban: 2,000–20,000 inhabitants; rural: <2,000 inhabitants.

The mean IgG avidity index was 75% (range 43%–100%), and there was no difference in the pattern of the avidity index distribution between IgG-positive/IgM-positive and IgG-positive/IgM-negative persons (supporting information Figure S2). No IgM antibodies against TBEV and DENV were detected in any of the 41 samples, while low-titre cross-reactivity was observed in IgG antibodies (data not shown).

The ratio of WNND patients to infected persons was found to be 1:141 (95% CI: 1:209–1:92). This ratio increased with age, and was considerably higher in persons aged 70 years or older (table 2); it was 1:266 (95% CI: 1:421–1:166) in 18–69 year olds, and 1:32 (95% CI: 1:56–1:18) in those aged ≥ 70 years. We estimated that approximately 28,000 (95% CI: 18,000–41,000) persons of all ages were infected in Greece during the 2010 epidemic.

**Table 2:** Ratio of West Nile neuroinvasive disease to infection by age-group.

**Age (years)**	**WNND cases in study area (surveillance data)**	**Population in study area (census data**) [24,25]	**Ratio of WNND to infection (95% CI)**
<18	2	40,064	NA
18–39	5	61,258	1:331 (1:870–1:123)
40–59	10	48,671	1:277 (1:492–1:151)
60–69	12	25,447	1:225 (1:380–1:129)
70–79	33	14,279	1:38 (1:66–1:21)
80+	18	4,295	1:21 (1:37–1:12)
All ages	80	194,014	1:141 (1:209–1:92)

WNND: West Nile neuroinvasive disease95% CI: 95% confidence intervalNA: non applicable

After sensitivity analysis, the seroprevalence we found was 5.1% (95% CI: 3.5–7.4) and the WNND-to-infection ratio 1:124 (95% CI: 1:179–1:85) in all ages, 1:241 (95% CI: 1:366–1:155) in persons aged 18–69, and 1:27 (95% CI: 1:51–1:14) in those aged ≥70 (see also table S3). In this analysis, we estimated that about 24,000 (95% CI: 17,000–35,000) infections occurred during the epidemic. 

### Occurrence of West Nile fever

Of the 41 IgG-positive persons, 20.2% reported experiencing three or more WNF indicator symptoms, as opposed to 3.7% of the 603 seronegative persons; this corresponds to an attributable risk of 16.5%. A similar pattern was seen for two or more symptoms (26.9% and 7.2% respectively; attributable risk 19.7%). Combining these two estimates, we deduced that approximately 18% of persons infected with WNV developed symptomatic disease (table S4).

### Factors associated with seropositivity

Several factors were found to be associated with IgG seropositivity on univariable analysis (table S5) among all factors investigated (table S6). Factors which remained significantly associated with IgG seropositivity in multivariable analysis comprised living in a rural/semi-urban area; being a housekeeper; being retired; having water repositories on one’s property; and agricultural labour. In the sub-analysis of rural/semi-urban data only, factors associated with IgG seropositivity were the presence of an irrigation drainage ditch >1km in length within a 500m radius of the household, being retired, being a housekeeper, and having water repositories on one’s property (table 3). No personal protective factors were found to be associated with seropositivity.

**Table 3:** Results of multivariable analyses: factors associated with IgG seropositivity for West Nile virus.

**Exposure variable**		Adjusted Prevalence Ratio (95% CI)*	**P-value**
***Analysis of full dataset (n=614)***			
**Area of residence†**	Urban	Reference	
	Rural/semi-urban	8.18 (1.14–58.72)	0.037
**Employment status**	Employed	Reference	
	Retired	2.60 (1.38–4·90)	0.003
	Housekeeper	4.30 (2·18–8.46)	<0.001
**Water repositories on property**	No	Reference	
	Yes	2.67 (1.43–5.00)	0.002
**Main profession**	Other	Reference	
	Agricultural labour	2.93 (1.34–6.40)	0.007
***Subanalysis from rural/semi-urban*^‡^*areas only* (*n=442*)**			
**Irrigation drainage ditch within 500 m radius**	No	Reference	
	Yes - ditch <1km in length	6.84 (0.74–63.13)	0.090
	Yes - ditch >1km in length	8.62 (1.02–73.18)	0.048
**Employment status**	Employed	Reference	
	Retired	3.20 (1.78–5.76)	<0.001
	Housekeeper	2.38 (1.04–5.44)	0.040
**Water repositories on property**	No	Reference	
	Yes	2.66 (1.26–5.64)	0.011

95% CI: 95% confidence interval

* Adjusted prevalence ratios and confidence intervals are weighted by age and urban/rural area of residence, and adjusted for cluster design.

† Area of residence: urban: >20,000 inhabitants; semi-urban: 2,000–20,000 inhabitants; rural: <2,000 inhabitants.

## Discussion

To our knowledge, this study is the first population seroprevalence survey following a WNV L2 epidemic. Our results show that the impact of the epidemic was much larger than suggested by the 262 cases notified to the public health authorities. The seroprevalence we found in the study area was 5.1–5.8%, and we estimated that 24,000–28,000 infections occurred nationally. We concluded that about 1 in 130 (1:141 to 1:124) infected persons of all ages developed WNND, and approximately 18% experienced symptoms typical of WNF. The outbreak had a rural character in the study area, with people living in less populated areas at an eight times higher risk for infection.

WNV IgG and IgM antibodies were detected in 15 participants, while another 26 had only IgG antibodies. The absence of IgM antibodies can be explained by the fact that the study was conducted 14–18 weeks after the epidemic peak, and the IgM antibodies had probably disappeared. This is in accordance with results of a recent study among Greek patients with WNV infection, which found that on average the IgM index becomes negative 164 (95% CI: 99–236) days after symptom onset [27]. The detection of high IgG indices among IgG-positive/IgM-negative persons (Figure S1), and the similar distribution of avidity indices in this group and the IgM-positive group (Figure S2) are suggestive of recent infection. Furthermore, after sensitivity analysis (in which IgG-positive participants with low IgG index were classified as IgG-negative), our estimate of seroprevalence was not substantially altered. Therefore, we believe that the estimated seroprevalence of 5.1% to 5.8% is due to the recent WNV L2 outbreak rather than past infection.

This is supported by evidence that WNV L2 was recently introduced to Greece [28] and the fact that the same WNV L2 strain, the only strain isolated in Greece since 2010, was molecularly detected in human clinical cases, blood donors, mosquitoes, wild birds and sentinel chickens on several occasions [7-12]. Furthermore, before this outbreak, no case of WNV clinical disease had ever been reported to the health authorities in Greece [6], although serological evidence suggests that low-level WNV circulation pre-existed (seroprevalence around 1% or less) [28-30]. The difference between our main seroprevalence estimate (5.8%) and the sensitivity analysis estimate (5.1%) was 0.7%, which is of the same magnitude as the WNV seroprevalence found before this outbreak, during the 2000s, in northern Greece (0.6–1.0%) [28,29].

Cross-reactivity in serology is common among flaviviruses. We found low-titre cross-reactivity in IgG antibodies between WNV and DENV, and to a lesser degree between WNV and TBEV, similar to findings of previous studies [31]. All WNV IgM- or IgG-positive samples were IgM-negative for TBEV and DENV. We did not conduct neutralisation tests to confirm WNV antibody results from ELISA. However, TBEV—though endemic in central and northern Europe—is not endemic in Greece, and DENV has not been detected in the country since 1928 [31]. Furthermore, patients diagnosed with WNV infection using ELISA during the outbreak who were then tested for neutralising antibodies all had positive results (n=14) [6]. Usutu virus, another mosquito-borne flavivirus reported recently in several European countries, has never been detected during studies on mosquitoes in northern Greece using generic flavivirus primers. The Greek goat encephalitis virus, a tick-borne flavivirus closely related to TBEV, was detected in two *Ixodes ricinus* ticks among 3,144 *Ixodidae* ticks collected during 2003–2006 from goats and sheep in rural areas of northern Greece, indicating limited circulation [32]. These data suggest that false-positive WNV antibody results due to cross-reactivity with other flavivurses are unlikely in the present study.

On univariable analysis, we found that seropositive persons tended to be older than seronegative ones, but age did not remain an independent risk factor for infection in the final multivariable model (table 3). This probably reflects aspects of lifestyle predisposing older people to mosquito exposure (e.g. outdoor activities, water repositories or animals in one’s property, less frequent use of insecticides etc.) rather than increased susceptibility to infection or increased chance of past infection in older persons. This is corroborated by our finding that the age pattern of IgG-positive/IgM-negative participants was similar to that of IgM-positive participants (table S2). Evidence of higher probability of exposure among older persons has been found in other WNV seroprevalence studies [22,33] as well as in a study carried out after a Chikungunya infection outbreak in Italy [34].

In the present study, estimates of WNND-to-infection ratios were based on a considerable number of WNND cases reported during the outbreak from the study area (n=80), unlike the respective estimates elsewhere [17]. This gave us a better opportunity to estimate ratios by age-group (table s2 and S3). We estimated that about 1 in 30 (1:32 to 1:27) infected persons aged 70 years or older developed WNND, as opposed to approximately 1 in 250 (1:266 to 1:241) adults aged 18–69 years. The observation that WNND-to-infection ratio increases with age has been consistently reported [1,17,20-22].

Our study demonstrated that approximately 18% of persons with WNV infection manifested symptoms attributable to this infection. Other surveys conducted after WNV L1 outbreaks have shown the respective proportion to be 20–25% [17,18]. The interval between presumed time of infection and time of data collection was about two months longer in our study compared to other surveys [17,18], which may have affected recall of symptoms, although introduction of bias is not expected as antibody status was unknown at the time of interview. This might have resulted in a degree of non-differential misclassification regarding WNF symptoms, thereby underestimating our respective findings.

As in previous WNV L1 studies [1,17,21-23], the risk groups we identified likely reflect increased exposure to mosquitoes and their breeding sites. The rural areas of Central Macedonia comprise approximately 20% of the wetlands in Greece, providing a favourable environment for mosquito reproduction [35]. Thus, it is unsurprising that the residents of rural/semi-urban areas and agricultural labourers had an eight and three times higher risk of infection, respectively. The higher risk of housekeepers and pensioners may be explained by the propensity in rural Greece for these two groups to spend more time at home and outdoors, often engaged in agricultural activities. Standing water, preferred by *Culex* mosquitoes, may be implicated in the higher risk of people with water repositories on their property, and of rural/semi-urban residents living close to an irrigation drainage ditch. 

In conclusion, the present study demonstrates that a WNV L2 strain prompted an epidemic essentially similar to previous WNV L1 outbreaks in terms of virulence, disease-to-infection ratio, and risk factors. Given the historic propensity for rapid geographical spread of WNV [1], the circulation of WNV L2 in parts of Europe [2,15,16], and the evidence of WNV L2 circulation in expanding areas in Greece [36,37], it can be expected that the public health impact of future WNV L2 outbreaks in Europe may be substantial. In the absence of specific treatment or a vaccine, efforts to reduce this impact should focus on mosquito control activities and public education, paying particular attention to groups with high risk of mosquito exposure and of severe disease. 

## Supporting Information

Figure S1 **Comparison of IgG index distribution in West Nile virus (WNV) IgG-positive/IgM-positive and IgG-positive/IgM-negative study participants.** Lower IgG indices can be expected in IgG-positive/IgM-negative participants compared to those who were IgM-positive, due to waning immunity in the former group. However, this tendency was not statistically significant in our data set, although within the IgG-positive/IgM-negative group there were four participants with low outlier values of IgG index. When these four outlier values are disregarded, minimum, 25th percentile, and median IgG indices in the IgM-negative group are in fact higher than in the IgM-positive group (see figure), with the minimum value (3.8) being substantially higher than the cut-off for a positive result (1.5). In the sensitivity analysis (table S3), we classified these four individuals with outlier IgG indices as IgG-negative. Note that: (1) Box depicts inter-quartile range (IQR); Adjacent values (whiskers) represent most extreme values within IQRx1.5 from the nearest quartile; Line within box represents median; Circles depicts outlier observations; (2) The criterion for positive result was IgG index >1.5; (3) Comparison of IgG index in the two groups: IgG index in the IgM-positive group is not significantly higher than in the IgM-negative group (Wilcoxon rank-sum (Mann-Whitney) test, one-tailed, p= 0.477).
(TIF)

Figure S2 **Comparison of West Nile virus (WNV) IgG avidity distribution in IgG-positive/IgM-positive and IgG-positive/IgM-negative study participants.** High avidity indices (>40%) were found in all cases, indicating that infection occurred more than 3 months before drawing blood samples. This can be explained by the long interval between epidemic and study implementation (14–18 weeks after the epidemic peak and 7–11 weeks after the last case was reported). Avidity indices in IgM-negative individuals were not higher that in those who were IgM-positive.
Note that: (1) Box represents inter-quartile range (IQR); Adjacent values (whiskers) represent most extreme values within IQRx1.5 from the nearest quartile; Line within box depicts median; Circles depict outlier observations; (2) The criterion for recent infection (i.e. less than 3 months) was IgG avidity index <40%; (3) Comparison of IgG avidity in the two groups: IgG avidity in the IgM-negative group is not significantly higher than in the IgM-positive group (Wilcoxon rank-sum (Mann-Whitney) test, one-tailed, p= 0.339).
(TIF)

Table S1 **Demographic characteristics of study participants and respective data for study area from census, 2001.**
(DOCX)

Table S2 **Mean age in different groups according to West Nile virus IgG and IgM status.** In the present study, WNV IgG-positive participants tended to be older than those who were IgG-negative (main text table 1). If this were related to older age in the IgG-positive/IgM-negative group compared to the IgM-positive group, this might suggest that persons in the former group had acquired immunity over a long period of time (with older persons therefore more likely to show evidence of infection). This would cast doubt on one of the important premises of the present study—that IgG-positive participants were infected during the 2010 outbreak (see Discussion in main text). To investigate this we compared mean age in different groups according to WNV IgG and IgM status (table). Mean age in the IgG-positive/IgM-negative group was not higher than that in the IgM-positive group (see table); in fact, the point-estimate of mean age was lower in the former group. Participants in all IgG-positive subgroups tended to be older than those who were IgG-negative (see table). We attributed this to lifestyle aspects predisposing older persons to mosquito exposure, a finding also detected elsewhere [22,33] (see Discussion in main text).
(DOCX)

Table S3 **Sensitivity analysis: West Nile virus IgG seroprevalence and ratio of West Nile neuroinvasive disease to infection by age group.**
(DOCX)

Table S4 **West Nile fever indicator symptoms in seropositive and seronegative study participants, prevalence ratios, and attributable risks.** Zou et al [18] identified eight indicator symptoms (those listed in the table, with myalgia and arthralgia recorded as two separate symptoms), and defined as cases of symptomatic West Nile infection persons with ≥3 indicator symptoms. For consistency in participants’ responses, we combined myalgia and arthralgia in one symptom (our study was carried out 14–18 weeks after the epidemic peak), and we estimated the proportion of persons manifesting West Nile fever by calculating the average of the risk of having ≥2 indicator symptoms attributable to WNV infection and that of having ≥3 indicator symptoms.
(DOCX)

Table S5 **Factors associated with IgG seropositivity for West Nile virus on univariable analysis.**
(DOCX)

Table S6 **Personal, household and local environmental information collected.**
(DOCX)
