# Supplementary figures and images for: Population Seroprevalence Study after a West Nile Virus Lineage 2 Epidemic, Greece, 2010
Source: PLoS One. 2013 Nov 18;8(11):e80432. doi: 10.1371/journal.pone.0080432 (PMC3832368; doi:10.1371/journal.pone.0080432)

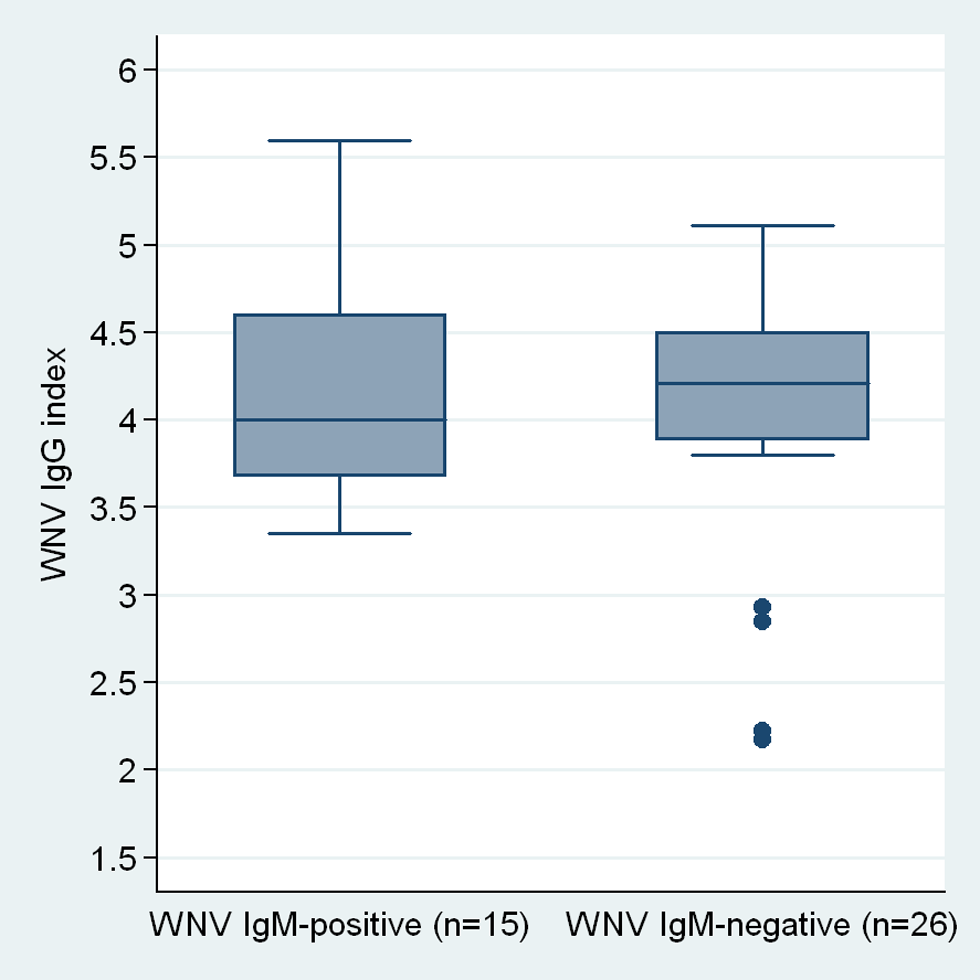

Supplement: Figure S1 — Comparison of IgG index distribution in West Nile virus (WNV) IgG-positive/IgM-positive and IgG-positive/IgM-negative study participants. Lower IgG indices can be expected in IgG-positive/IgM-negative participants compared to those who were IgM-positive, due to waning immunity in the former group. However, this tendency was not statistically significant in our data set, although within the IgG-positive/IgM-negative group there were four participants with low outlier values of IgG index. When these four outlier values are disregarded, minimum, 25th percentile, and median IgG indices in the IgM-negative group are in fact higher than in the IgM-positive group (see figure), with the minimum value (3.8) being substantially higher than the cut-off for a positive result (1.5). In the sensitivity analysis (table S3), we classified these four individuals with outlier IgG indices as IgG-negative. Note that: (1) Box depicts inter-quartile range (IQR); Adjacent values (whiskers) represent most extreme values within IQRx1.5 from the nearest quartile; Line within box represents median; Circles depicts outlier observations; (2) The criterion for positive result was IgG index >1.5; (3) Comparison of IgG index in the two groups: IgG index in the IgM-positive group is not significantly higher than in the IgM-negative group (Wilcoxon rank-sum (Mann-Whitney) test, one-tailed, p= 0.477). (TIF) [file pone.0080432.s001.tif]

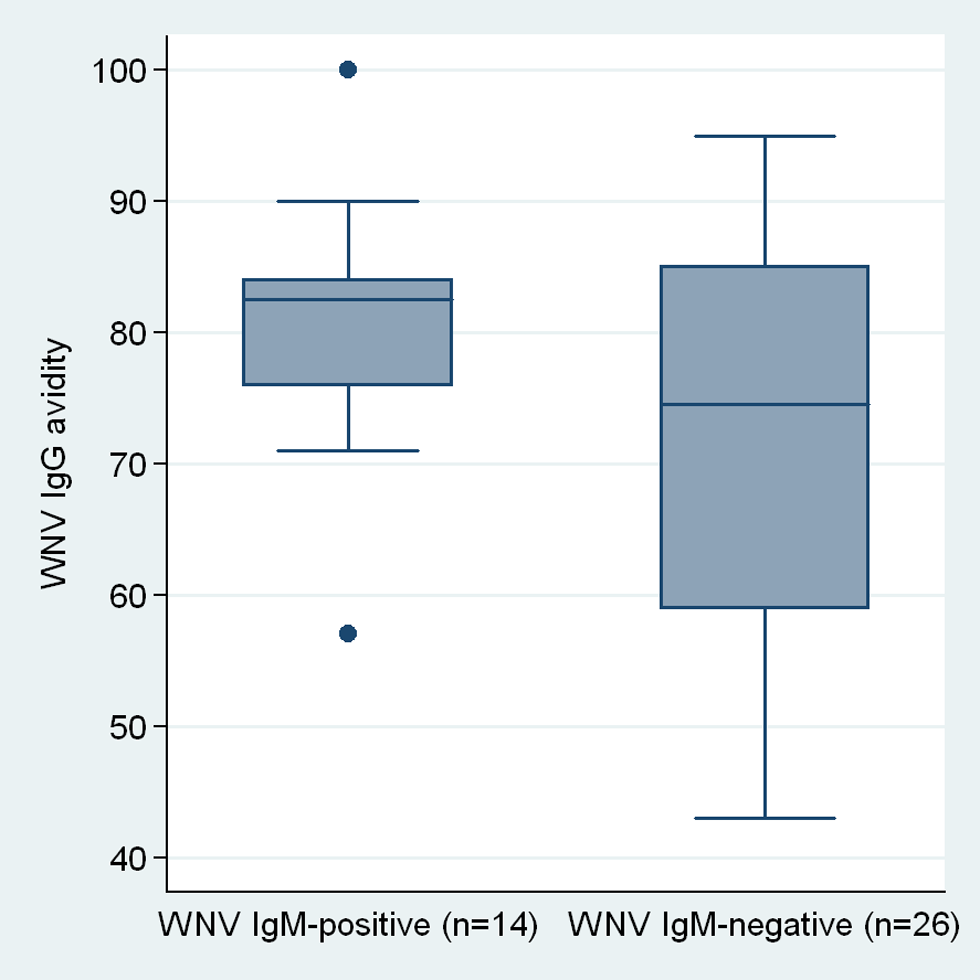

Supplement: Figure S2 — Comparison of West Nile virus (WNV) IgG avidity distribution in IgG-positive/IgM-positive and IgG-positive/IgM-negative study participants. High avidity indices (>40%) were found in all cases, indicating that infection occurred more than 3 months before drawing blood samples. This can be explained by the long interval between epidemic and study implementation (14–18 weeks after the epidemic peak and 7–11 weeks after the last case was reported). Avidity indices in IgM-negative individuals were not higher that in those who were IgM-positive. Note that: (1) Box represents inter-quartile range (IQR); Adjacent values (whiskers) represent most extreme values within IQRx1.5 from the nearest quartile; Line within box depicts median; Circles depict outlier observations; (2) The criterion for recent infection (i.e. less than 3 months) was IgG avidity index <40%; (3) Comparison of IgG avidity in the two groups: IgG avidity in the IgM-negative group is not significantly higher than in the IgM-positive group (Wilcoxon rank-sum (Mann-Whitney) test, one-tailed, p= 0.339). (TIF) [file pone.0080432.s002.tif]
