# Supplementary material for: Population Seroprevalence Study after a West Nile Virus Lineage 2 Epidemic, Greece, 2010
Source: PLoS One. 2013 Nov 18;8(11):e80432. doi: 10.1371/journal.pone.0080432 (PMC3832368; doi:10.1371/journal.pone.0080432)
Supplement: Table S1 — Demographic characteristics of study participants and respective data for study area from census, 2001. (DOCX) [file pone.0080432.s003.docx]

|  |  | **Study participants*** | | **Census data†** |
| --- | --- | --- | --- | --- |
|  |  | **N** | **%** | **%** |
| **Gender** | Female | 388 | 53.7 | 50.2 |
|  | Male | 335 | 46.3 | 49.8 |
|  | Total | 723 | 100 | 100 |
| **Age (years)** | 18-29 | 79 | 10.9 | 21.5 |
|  | 30-39 | 109 | 15.1 | 18.3 |
|  | 40-49 | 146 | 20.2 | 16.9 |
|  | 50-59 | 126 | 17.4 | 14.7 |
|  | 60-69 | 112 | 15.5 | 16.5 |
|  | 70-79 | 122 | 16.9 | 9.3 |
|  | 80+ | 29 | 4.0 | 2.8 |
|  | Total (≥18 years) | 723 | 100 | 100 |
| **Area of residence‡** | Urban | 232 | 32.1 | 37.1 |
|  | Semi-urban | 215 | 29.7 | 23.3 |
|  | Rural | 276 | 38.2 | 39.7 |
|  | Total | 723 | 100 | 100 |

*Study participant proportions are not weighted.

†Census data refer to the study area; data for age and area of residence pertain to population aged ≥18 years (n=153,950), and data for gender to all ages (n=194,014) [24,25].

‡Area of residence: urban: >20,000 inhabitants; semi-urban: 2,000–20,000 inhabitants; rural: <2,000 inhabitants.
