# Supplementary material for: Population Seroprevalence Study after a West Nile Virus Lineage 2 Epidemic, Greece, 2010
Source: PLoS One. 2013 Nov 18;8(11):e80432. doi: 10.1371/journal.pone.0080432 (PMC3832368; doi:10.1371/journal.pone.0080432)
Supplement: Table S2 — Mean age in different groups according to West Nile virus IgG and IgM status. In the present study, WNV IgG-positive participants tended to be older than those who were IgG-negative (main text table 1). If this were related to older age in the IgG-positive/IgM-negative group compared to the IgM-positive group, this might suggest that persons in the former group had acquired immunity over a long period of time (with older persons therefore more likely to show evidence of infection). This would cast doubt on one of the important premises of the present study—that IgG-positive participants were infected during the 2010 outbreak (see Discussion in main text). To investigate this we compared mean age in different groups according to WNV IgG and IgM status (table). Mean age in the IgG-positive/IgM-negative group was not higher than that in the IgM-positive group (see table); in fact, the point-estimate of mean age was lower in the former group. Participants in all IgG-positive subgroups tended to be older than those who were IgG-negative (see table). We attributed this to lifestyle aspects predisposing older persons to mosquito exposure, a finding also detected elsewhere [22,33] (see Discussion in main text). (DOCX) [file pone.0080432.s004.docx]

|  |  | **Age (years)** | |  |  |
| --- | --- | --- | --- | --- | --- |
|  | **N** | **Mean** | **95% CI** | **t-statistic** | **P-value** |
| WNV IgG-negative | 603 | 52.2 | 50.9–52.2 | Reference |  |
| WNV IgG-positive/IgM-negative* | 26 | 58.5 | 51.7–65.4 | -1.8310 | 0.068 |
| WNV IgG-positive/IgM-positive*† | 15 | 63.1 | 55.1–71.0 | -2.4191 | 0.016 |
| WNV IgG-positive (all) | 41 | 60.2 | 55.1–65.2 | -2.8805 | 0.004 |

WNV: West Nile virus

95% CI: 95% confidence interval

*Comparison of age in the IgG-positive/IgM-negative group and the IgG-positive/IgM-positive group: mean age in the former group is not significantly higher than in the latter one (t-test, one-tailed, t=-0·8705, p= 0·805).

†All WNV IgM-positive individuals were also WNV IgG-positive.
