# Supplementary material for: Population Seroprevalence Study after a West Nile Virus Lineage 2 Epidemic, Greece, 2010
Source: PLoS One. 2013 Nov 18;8(11):e80432. doi: 10.1371/journal.pone.0080432 (PMC3832368; doi:10.1371/journal.pone.0080432)
Supplement: Table S3 — Sensitivity analysis: West Nile virus IgG seroprevalence and ratio of West Nile neuroinvasive disease to infection by age group. (DOCX) [file pone.0080432.s005.docx]

|  | **WNV IgG-positive*†** | |  |
| --- | --- | --- | --- |
| **Age (years)** | **n/N** | **% (95% CI)** | **Ratio of WNND to infection (95% CI)*** |
| 18-39 | 3/161 | 1.9 (0.6–5.9) | 1:233 (1:723−1:74) |
| 40-59 | 14/238 | 5.7 (3.1–10.1) | 1:277 (1:492–1:151) |
| 60-69 | 10/103 | 9.6 (5.2–17.0) | 1:204 (1:360–1:110) |
| 70-79‡ |  |  | 1:32 (1:60–1:16) |
| 80+‡ |  |  | 1:17 (1:33–1:9) |
| All ages | 37/644 | 5.1 (3.5–7.4) | 1:124 (1:179–1:85) |

WNV: West Nile virus

WNND: West Nile neuroinvasive disease

95% CI: 95% confidence interval

*In this analysis, individuals who were IgG-positive/IgM-negative and had low outlier values of IgG index were classified as IgG-negative (n=4, see figure S1).

†Proportions and their confidence intervals are weighted by age and urban/rural area of residence, and adjusted for cluster design.

‡WNV IgG-positive individuals in the 70+ years age-group: 10/142; weighted proportion: 7.3% (95% CI: 3.7–13.9%).
