# Supplementary material for: Population Seroprevalence Study after a West Nile Virus Lineage 2 Epidemic, Greece, 2010
Source: PLoS One. 2013 Nov 18;8(11):e80432. doi: 10.1371/journal.pone.0080432 (PMC3832368; doi:10.1371/journal.pone.0080432)
Supplement: Table S4 — West Nile fever indicator symptoms in seropositive and seronegative study participants, prevalence ratios, and attributable risks. Zou et al [18] identified eight indicator symptoms (those listed in the table, with myalgia and arthralgia recorded as two separate symptoms), and defined as cases of symptomatic West Nile infection persons with ≥3 indicator symptoms. For consistency in participants’ responses, we combined myalgia and arthralgia in one symptom (our study was carried out 14–18 weeks after the epidemic peak), and we estimated the proportion of persons manifesting West Nile fever by calculating the average of the risk of having ≥2 indicator symptoms attributable to WNV infection and that of having ≥3 indicator symptoms. (DOCX) [file pone.0080432.s006.docx]

|  | **WNV IgG positive**  **(n=41)** | | **WNV IgG negative**  **(n=603)** | |  |  |
| --- | --- | --- | --- | --- | --- | --- |
| **West Nile fever**  **indicator symptoms*** | **N** | **% (95% CI)†** | **N** | **% (95% CI)†** | **Prevalence ratio**  **(95% CI)†** | **Attributable risk**  **% (95% CI)†** |
| Myalgia/arthralgia | 9 | 24.7 (13.2–41.4) | 70 | 11.7 (8.7–15.6) | 2.1 (1.2–3.8) | 13.0 (-1.4–27.4) |
| Headache | 8 | 20.8 (9.9–38.7) | 83 | 14.6 (11.7–18.1) | 1.4 (0.7–2.9) | 6.2 (-8.3–20.7) |
| Generalised weakness | 8 | 19.1 (9.4–35.0) | 28 | 4.8 (3.1–7.5) | 4.0 (1.9–8.3) | 14.3 (1.6–27.0) |
| Skin rash | 8 | 21.9 (8.2–46.8) | 50 | 8.9 (6.6–11.6) | 2.5 (1.0–6.3) | 13.0 (-6.3–32.3) |
| Fever | 7 | 17.6 (7.8–35.1) | 39 | 7.4 (5.0–10.7) | 2.4 (1.0–5.5) | 10.2 (-3.3–23.7) |
| Chills | 6 | 16.3 (6.7–34.5) | 41 | 7.2 (4.9–10.2) | 2.3 (1.0–5.3) | 9.1 (-4.4–22.6) |
| Ocular pain | 2 | 3.9 (1.0–14.3) | 19 | 3.5 (2.2–5.5) | 1.1 (0.3–4.6) | 0.4 (-5.0–5.8) |
| ≥ 2 symptoms | 11 | 26.9 (15.3–42.8) | 37 | 7.2 (5.1–10.2) | 3.7 (2.0–6.9) | 19.7 (5.8–33.6) |
| ≥ 3 symptoms | 8 | 20.2 (9.8–36.9) | 20 | 3.7 (2.1–6.2) | 5.5 (2.4–12.5) | 16.5 (3.1–29.9) |

WNV: West Nile virus

95% CI: 95% confidence interval

*West Nile fever indicator symptoms modified from Zou et al 2010 [18].

†Proportions, prevalence ratios, and their confidence intervals are weighted by age and urban/rural area of residence, and adjusted for cluster design.
