# Supplementary material for: Population Seroprevalence Study after a West Nile Virus Lineage 2 Epidemic, Greece, 2010
Source: PLoS One. 2013 Nov 18;8(11):e80432. doi: 10.1371/journal.pone.0080432 (PMC3832368; doi:10.1371/journal.pone.0080432)
Supplement: Table S5 — Factors associated with IgG seropositivity for West Nile virus on univariable analysis. (DOCX) [file pone.0080432.s007.docx]

|  |  | **Total sample** | | IgG-positive | | Prevalence ratio |
| --- | --- | --- | --- | --- | --- | --- |
| Exposure variable |  | **N** | **% (95% CI)*** | **N** | % (95% CI)* | % (95% CI)* |
| **Age-group** | <60 years | 460 | 71.4 (67.6–74.9) | 18 | 4.0 (2.2–7.3) | Reference |
|  | 60 years or over | 263 | 28.6 (25.1–32.4) | 23 | 9.8 (6.5–14.6) | 2.4 (1.3–4.6) |
|  | Total | 723 |  | 41 |  |  |
| **Area of residence†** | Urban | 232 | 37.1 (25.1–50.8) | 3 | 1.4 (0.3–6.9) | Reference |
|  | Semi-urban | 215 | 23.3 (14.3–35.5) | 12 | 5.3 (2.8–9.8) | 3.8 (0.7–21.7) |
|  | Rural | 276 | 39.7 (27.3–53.4) | 26 | 10.0 (6.5–15.3) | 7.3 (1.4–38.8) |
|  | Total | 723 |  | 41 |  |  |
| **Education level** | Further education (>12 years) | 324 | 51.7 (45.6–57.7) | 9 | 2.2 (0.9–5.3) | Reference |
|  | High school graduate (12 years) or lower | 399 | 48.3 (42.3–54.4) | 32 | 9.5 (6.3–14.1) | 4.3 (1.7–11.1) |
|  | Total | 723 |  | 41 |  |  |
| **Employment status** | Employed/other occupation | 392 | 61.8 (58.0–65.5) | 10 | 2.8 (1.5–5.2) | Reference |
|  | Retired | 189 | 20.1 (17.3–23.3) | 19 | 11.6 (7.1–18.3) | 4.1 (2.3–7.3) |
|  | Housekeeper | 141 | 18.0 (15.3–21.1) | 12 | 8.9 (4.6–16.5) | 3.2 (1.4–7.2) |
|  | Total | 722 |  | 41 |  |  |
| **Main profession** | Other | 522 | 77.7 (72.0–82.5) | 20 | 3.7 (2.2–6.2) | Reference |
|  | Agricultural labour | 168 | 22.3 (17.5–28.0) | 18 | 12.3 (6.8–21.3) | 3.3 (1.6–7.0) |
|  | Total | 690 |  | 38 |  |  |
| **Agricultural labour‡** | No | 250 | 59.2 (50.8–67.1) | 3 | 1.1 (0.3–3.5) | Reference |
|  | Yes | 193 | 40.8 (32.9–49.2) | 16 | 9.4 (5.4–15.9) | 8.6 (2.6–28.6) |
|  | Total | 443 |  | 19 |  |  |
| **Activities with animals** | No | 575 | 80.8 (75.7–85.0) | 25 | 4.7 (2.9–7.4) | Reference |
|  | Yes | 146 | 19.2 (15.0–24.3) | 16 | 10.3 (6.1–16.7) | 2.2 (1.2–4.0) |
|  | Total | 721 |  | 41 |  |  |
| **Gardening actitivies** | Less than once a week/never | 313 | 49.7 (45.0–54.5) | 8 | 3.4 (1.5–7.3) | Reference |
|  | Once a week or more | 398 | 50.3(45.5–55.0) | 33 | 8.2 (5.6–11.8) | 2.4 (1.2–5.0) |
|  | Total | 711 |  | 41 |  |  |
| **Avoided going out at night** | No | 586 | 84.2 (80.1–87.7) | 27 | 4.8 (2.9–7.9) | Reference |
|  | Yes | 136 | 15.8 (12.3–19.9) | 14 | 10.7 (6.6–16.9) | 2.2 (1.2–4.2) |
|  | Total | 722 |  | 41 |  |  |
| **Indoor use of insecticide spray** | No | 480 | 66.7 (60.5–72.4) | 20 | 4.3 (2.6–7.0) | Reference |
|  | Yes | 242 | 33.3 (27.6–39.5) | 21 | 8.8 (5.4–13.9) | 2.1 (1.2–3.7) |
|  | Total | 722 |  | 41 |  |  |
| **Water repositories on property** | No | 659 | 90.1 (83.1–94.4) | 31 | 4.6 (3.0–7.0) | Reference |
|  | Yes | 64 | 9.9 (5.6–16.9) | 10 | 16.1 (7.5–31.1) | 3.5 (1.5–8.0) |
|  | Total | 723 |  | 41 |  |  |
| **Farm animals on property** | No | 677 | 94.5 (87.2–97.7) | 34 | 5.0 (3.3–7.5) | Reference |
|  | Yes | 39 | 5.5 (2.3–12.8) | 6 | 16.8 (6.5–36.9) | 3.4 (1.4–8.4) |
|  | Total | 716 |  | 40 |  |  |
| **Presence of screens on windows** | None/some | 275 | 40.2 (32.8–48.0) | 8 | 2.2 (0.9–5.0) | Reference |
|  | Yes, all of the windows | 440 | 59.8 (52.0–67.2) | 31 | 7.7 (4.8–11.9) | 3.6 (1.4–9.1) |
|  | Total | 715 |  | 39 |  |  |

95% CI: 95% confidence interval

*Proportions, prevalence ratios, and their confidence intervals are weighted by age and urban/rural area of residence, and adjusted for cluster design

†Area of residence: urban: >20,000 inhabitants; semi-urban: 2,000–20,000 inhabitants; rural: <2,000 inhabitants.

‡Agricultural labour as main or secondary profession.
