# Supplementary material for: Population Seroprevalence Study after a West Nile Virus Lineage 2 Epidemic, Greece, 2010
Source: PLoS One. 2013 Nov 18;8(11):e80432. doi: 10.1371/journal.pone.0080432 (PMC3832368; doi:10.1371/journal.pone.0080432)
Supplement: Table S6 — Personal, household and local environmental information collected. (DOCX) [file pone.0080432.s008.docx]

| **Demographic characteristics** | Age |
| --- | --- |
|  | Gender |
|  | Place of residence |
|  | Ethnic minority |
|  | Education |
|  | Employment status |
|  | Occupation |
| **Personal risk factors: Outdoor activities** | Outdoor occupation |
|  | Agricultural main occupation |
|  | Agricultural activities |
|  | Activities with animals |
|  | Gardening activities |
|  | Habit to go fishing |
|  | Habit to go hunting |
|  | Habit to go hiking |
|  | Habit to go swimming |
|  | Sport activities outdoors |
|  | Habit to sit in open-air (balcony or terrace) |
|  | Hours spent outdoors at dusk/dawn |
|  | Saw dead birds |
| **Personal risk factors: Exposure to mosquitoes** | Ever bitten by mosquitoes |
|  | Felt mosquito bites (at dawn, daytime, dusk, evening) |
|  | Felt mosquito bites during sleep |
|  | Month most bitten by mosquitoes (June to September) |
|  | Habit to sleep outdoors |
|  | Habit to sleep with windows open (without screens) |
| **Personal risk factors: Protective measures** | Avoided going out at dusk |
|  | Avoided going out at night |
|  | Wore long sleeves and trousers |
|  | Took care to remove standing water from property |
|  | Used mosquito repellent |
|  | Used indoor anti-mosquito tablets or oil burners |
|  | Used indoor insecticide spray |
|  | Screens on windows of bedroom |
|  | Slept with air conditioning on |
|  | Slept with fan on (standing/ceiling fan) |
|  | Used bed net |
|  | Changed habits after WNV outbreak was known |
| **Personal medical history** | Condition requiring regular medical attention/treatment |
|  | Hypertension |
|  | Diabetes |
|  | Stroke |
|  | Heart condition |
|  | Respiratory condition |
|  | Kidney condition |
|  | Immune system condition |
|  | Cancer, lymphoma or leukaemia |
|  | Takes medication regularly for health problem |
|  | Takes immunosuppressive medication |
|  | Smoking habits |
| **Household risk factors: General** | Total number of persons living in household |
|  | Type of housing |
|  | Property has garden/outdoor space |
|  | Type of sewage system |
| **Household risk factors: Protective measures** | Property has window screens |
|  | Property has air conditioning |
|  | Property has fan (floor/ceiling fan) |
| **Household risk factors: Water containers** | Flowerpots |
|  | Bird baths |
|  | Cistern |
|  | Swimming pool |
|  | Other water container |
| **Household risk factors: Plants in garden/property** | Vegetables planted |
|  | Corn planted |
|  | Rice planted |
| **Household risk factors: Animals/birds in property** | Dogs/cats |
|  | Sheep/goats |
|  | Other mammals (horses, pigs etc.) |
|  | Chickens/ducks/geese |
|  | Pigeons |
|  | Birds in a cage |
| **Local environmental risk factors: Water surfaces*** | Number of rainwater drain points within 50m/100m |
|  | Length of rainwater channels within 50m/100m |
|  | Length of irrigation drainage ditches within 500m |
|  | Surface of flooded area within 500m |
| **Local environmental risk factors: Wastewater*** | Number of cesspits within 50m/100m |
|  | Distance from wastewater treatment plant (within 1km) |
| **Local environmental risk factors: Animals/birds** | Number of sheep/livestock units within 1km |
|  | Number of animal breeding facilities within 50m/100m |
|  | Number of chicken sheds within 50m/100m |
|  | Number of duck/geese sheds within 100m |
|  | Number of pigeon houses within 50m/100m |
|  | Number of stork nests within 100m |

*Distances included are distance from household.
